# Supplementary material for: Association of Habitual Diet Quality and Nutrient Intake with Cognitive Performance in Community-Dwelling Older Adults: A Cross-Sectional Study
Source: Nutrients. 2025 Sep 30;17(19):3139. doi: 10.3390/nu17193139 (PMC12525846; doi:10.3390/nu17193139)
Supplement: Supplementary file 1 [file nutrients-17-03139-s001.zip › nutrients-3868286-supplementary.pdf]

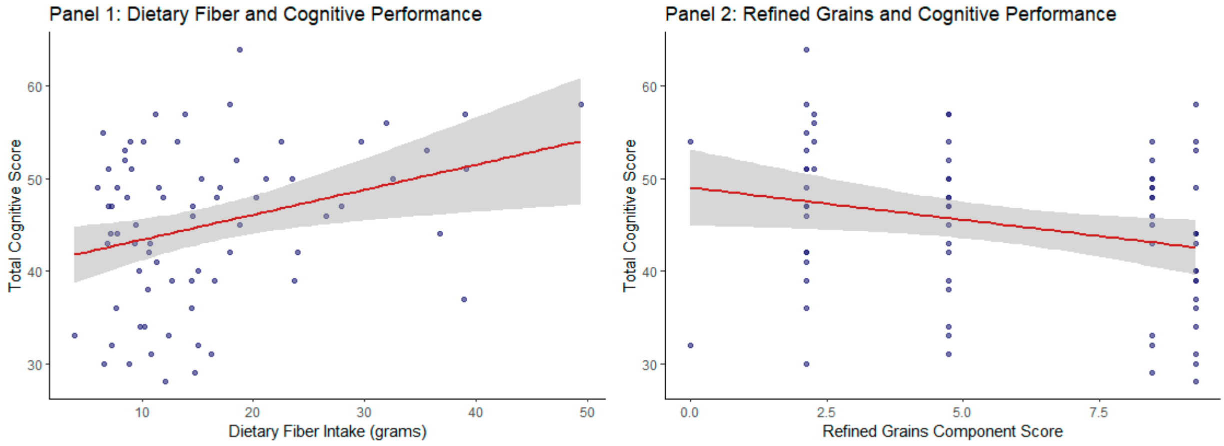

Figure S1. Associations of dietary fiber and refined grains with cognitive performance. Panel 1 illustrates the positive linear association between dietary fiber intake and total cognitive score (p=0.007). **Panel 2** illustrates the negative linear association between the refined grains component score and total cognitive score (p=0.011). In both panels, each point represents an individual participant. The solid red line indicates the line of best fit from a linear regression model, and the shaded area represents the 95% confidence interval.
